# Automatic classification of ICA components from infant EEG using MARA

**DOI:** 10.1016/j.dcn.2021.101024

**Published:** 2021-10-20

**Authors:** I. Marriott Haresign, E. Phillips, M. Whitehorn, V. Noreika, E.J.H. Jones, V. Leong, S.V. Wass

**Affiliations:** a Department of Psychology, University of East London, London, UK; b Department of Biological and Experimental Psychology, School of Biological and Chemical Sciences, Queen Mary University of London, UK; c Centre for Brain and Cognitive Development, Birkbeck College, University of London, UK; d Department of Experimental Psychology, University of Cambridge, Cambridge, UK; e School of Social Sciences, Nanyang Technological University, Singapore

**Keywords:** EEG, Deep learning, Artifact correction, Independent component analysis (ICA), Event-related potentials (ERP)

## Abstract

Automated systems for identifying and removing non-neural ICA components are growing in popularity among EEG researchers of adult populations. Infant EEG data differs in many ways from adult EEG data, but there exists almost no specific system for automated classification of source components from paediatric populations. Here, we adapt one of the most popular systems for adult ICA component classification for use with infant EEG data. Our adapted classifier significantly outperformed the original adult classifier on samples of naturalistic free play EEG data recorded from 10 to 12-month-old infants, achieving agreement rates with the manual classification of over 75% across two validation studies (n = 44, n = 25). Additionally, we examined both classifiers’ ability to remove stereotyped ocular artifact from a basic visual processing ERP dataset compared to manual ICA data cleaning. Here, the new classifier performed on level with expert manual cleaning and was again significantly better than the adult classifier at removing artifact whilst retaining a greater amount of genuine neural signal operationalised through comparing ERP activations in time and space. Our new system (iMARA) offers developmental EEG researchers a flexible tool for automatic identification and removal of artifactual ICA components.

## Highlights

• Currently, few tools are available for ICA based data correction that are designed around infant EEG.
• ICA correction is necessary for naturalistic paradigms when movement artifacts covary with cognitive processes.
• We present a system for automated ICA classification designed around infant EEG data.
• This system offers increased performance compared to systems of automatic ICA classification designed around adult EEG.

## 1 Introduction

The use of EEG in developmental cognitive neuroscience has led to a rich understanding of how the brain develops throughout early life. EEG has provided insights from birth into the development of skills such as face processing (e.g., Farroni et al., 2002), attention (e.g., Xie et al., 2018), memory (e.g., Jones et al., 2020) and social interaction (e.g., Wass et al., 2018). It has also been pivotal in identifying risk factors associated with developmental disorders (e.g., Orekhova et al., 2014) and later emerging psychopathology (e.g., Jones and Johnson, 2017). However, the field is challenged by a lack of scalable, standardised tools for artifact correction. In this paper, we present one ‘lossless’ approach for artifact correction tuned for infant EEG data.

### 1.1 Traditional approaches to artifact removal

Despite its value, EEG recorded from paediatric populations is particularly susceptible to artifact contamination. Furthermore, it typically contains fewer sections of clean uninterrupted data due to lower recording tolerances (Gabard-Durnam et al., 2018, Debnath et al., 2020). One common approach to combat this is to manually remove sections of the continuous data contaminated with artifact. However, this method of data cleaning can be problematic. For example, artifact correction in large EEG datasets can be very time consuming, and as developmental neuroscience is growing and EEG datasets are becoming larger, automated pre-processing tools are needed to efficiently process large-scale data, taking less time than manual cleaning (Webb et al., 2015). Further manual cleaning is inherently subjective and there exist few comprehensive reviews to guide researchers (e.g., Chaumon et al., 2015). Recent studies have introduced methods for automatically identifying and removing segments of data contaminated by artifact in paediatric populations (e.g., Gabard-Durnam et al., 2018). These types of studies address the need for standardisation and speed but often rely on complete removal of artifact-affected segments. Further, many of the currently available methods for paediatric EEG have procedures designed specifically for higher electrode density recordings, therefore it is also necessary to develop artifact correction approaches that are also flexible to low-density recordings, which are often used in infant EEG studies.

Recently, there has been a drive towards the use of more naturalistic paradigms in EEG research (Risko et al., 2016, Wass et al., 2020, Holleman et al., 2020). However, naturalistic EEG recordings provide additional analytical challenges over traditional screen-based tasks. For example, in traditional screen-based/ event-related tasks in which the child is passively exposed to a set of stimuli, artifacts are more randomly distributed with respect to simulation. Removal of sections containing significant artifact can in this context be potentially beneficial, as visual experience during these sections might also be different (e.g., at its simplest the child might be fussing and not be attending to the image on the screen). However, in naturalistic paradigms, removal of whole sections of data is particularly problematic because data segments contaminated by artifact often covary with cognitive/ attentional processes of interest. Specifically, in naturalistic paradigms, the 'simulation' is often child-controlled (e.g., the child turning to the parent in a naturalistic interaction), and so artifacts are more likely to be time-locked to neural signals of interest; the removal of artifact is thus likely to also affect the analysis of neural signals. Thus, we need approaches to the correction of artifact that remove artifactual signals from the EEG recording throughout the session, rather than removing whole segments of both signal and noise – so-called lossless pipelines.

### 1.2 Lossless approaches

Independent components analysis (ICA) is an alternative method that can be used to remove artifact from EEG data. When applied to EEG data, ICA separates the contributing sources to the scalp EEG into additive subcomponents, with varying contributions to the overall signal (Rutledge and Bouveresse, 2013, Makeig et al., 1996). Each ICA component typically contains a varying mix of neural and artifactual signals. Consideration of each component’s time-frequency and topographical properties forms the basis of manual ICA classification (e.g., Chaumon et al., 2015; see also appendix B and SM Fig. 1), which is typically used to separate the ICA components into two groups; components containing mostly artifactual signals and components containing mostly neural signals. As ICA itself is not a perfect method, in practice each component typically contains a varying amount of neural and artifactual signals. Some components can be clearly and easily identified manually as containing predominantly artifactual signals, whereas in other cases the mix of neural and artifactual signals is less clear and manual classification of these components becomes more subjective and based on the user’s experience level.

**Fig. 1:**
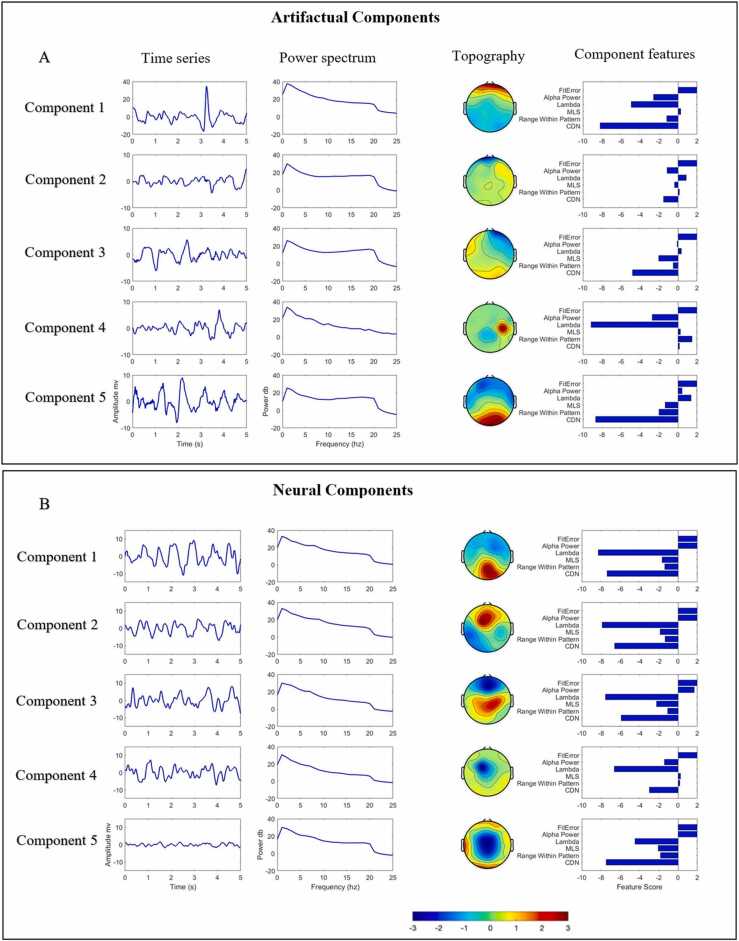
Examples (taken from the present study) of neural and artifactual ICA components identified by iMARA. A) Examples of components identified as ‘artifact’ by iMARA. B) Examples of components identified as ‘neural’ by iMARA. For both, the first column shows five-second segments of the components time course; the second shows the component power spectral density; the third shows the topographical activations; and the fourth their scores for the six features used in classification. Detailed descriptions of the six features are given in Appendix A.

ICA used in this way as a data preparation tool is often favoured by researchers because it allows them to subtract/ remove unwanted components (e.g., those associated with artifact) from the EEG data without reducing the overall amount of data (hence is lossless). This is a major advantage when compared to, for example, using amplitude thresholds to remove entire sections/trials of data that are contaminated by artifact. This is particularly true for naturalistic paradigms, for the reasons given above.

We note only one other attempt to provide a system for automatic ICA classification appropriate for paediatric EEG data. The adjusted-ADJUST system (Leach et al., 2020) provides developmental researchers with an excellent framework for automatic ICA classification from typical repeated stimulus EEG data. Leach and colleagues’ system achieved classification agreement with human coders of > 85% with EEG recorded from 6-month-old infants. Whilst this is an impressive system, it is limited in some ways in which iMARA is not. Firstly, the adjusted-ADJUST program is set up to primarily deal with stereotypical eye movement artifact. Three of the five categories it sorts ICA components into are related to ocular motor activity. iMARA was trained on over 600 ICA components, including a wide variety of stereotyped and non-stereotyped artifacts, and so is potentially more generalisable to a wider range of artifacts. Second, adjusted-ADJUST is designed for event-locked paradigms with a repeated stimulus and is not able to incorporate EEG data from continuous/ non-event locked paradigms, which are frequently used within developmental research (e.g., to study neural entrainment in parent-infant interactions (Wass et al., 2020),), whereas iMARA is flexible to data in either format. Overall, both systems perform well and depending on the data/situation one might be more optimal than the other.

### 1.3 The MARA classification system

Many researchers manually identify which ICA-components are associated with genuine neural activity, and which are artifact. Recently, however, there have been attempts to automate this process. In this paper, we focus on one **automated** method, the Multiple Artifact Rejection Algorithm (MARA) (Winkler et al., 2011). The MARA classification system is grounded in the use of a binary linear classifier, following:

(1) H=sign(w·x+b){−1,1},

Where w is a weight vector obtained from samples of labelled training data, x is a feature vector containing the values of all the different component features (as illustrated in Fig. 1) and b is a bias term. In short, this identifies which group the input data belong to. In the context of the MARA system, it classifies ICA components as either belonging to the ‘neural’ or ‘artifact’ group.

Classification also depends on the training data that is used. The MARA classifier was originally trained using 690 ICA-components (from an adult EEG reaction time study (n = 23 datasets)), which were manually classified as either ‘neural’ or ‘artifact. The accuracy of the classifier was then tested on 1080 additional components from the same study. Accuracy was tested by comparing the results of the automated ICA classification to manual ICA classification. The system achieved agreement rates of approximately 91%, (i.e., 9% of components were classified differently when comparing the automated and manual classification). Accuracy was then further tested on new data from two other studies; an auditory event-related potential (ERP) paradigm (n = 18 datasets); and a motor imagery BCI paradigm (n = 80 datasets), both with different channel setups and participants. Testing the performance of the classifier on the additional data revealed agreement/error rates between the automatic and manual classification of 85/15% (Winkler et al., 2011).

Despite its popularity within adult EEG research, MARA has not received much attention within paediatric EEG research. This is perhaps because ICA itself is not widely used within traditional paediatric ERP research as a pre-processing tool. One previous study quantified the performance of MARA with paediatric EEG data. Gabard-Durnham and colleagues incorporated the classifier as part of their pre-processing tool kit (HAPPE) (Gabard-Durnam et al., 2018), applying it to samples of high density (128 channels) resting-state EEG from infants and children aged 3–36 months. The authors found that when MARA was used in conjunction with ‘non-standard’ approaches (e.g., wavelet thresholding of the ICA), it rejected 42% of components, but when used as part of a ‘standard’ pre-processing pipeline e.g., including referencing, filtering, channel rejection/interpolation, trial/ continuous data rejection and omitting the wavelet thresholding step, MARA rejected over 85% of the components. These high rejection rates highlight the importance of retraining MARA with infant data – as, typically, researchers minimise the number of components rejected to preserve as much of the original data as possible. In the present study, we aim to address the need for systems for automatic ICA cleaning of infant EEG data that can be incorporated among other standard pre-processing procedures.

### 1.4 The need to tune artifact-removal approaches to infant EEG data

Infant EEG has unique properties, requiring the design of specific tools for processing. EEG recorded from infants differs from that of children (Lepage and Théoret, 2006) and adults (Stroganova et al., 1999). For example, the canonical frequency bands e.g., delta (1–4 Hz), theta (4–8 Hz), alpha (9–13 Hz), etc observed in adult EEG are observed at lower frequencies in infant EEG (Orekhova et al., 2006). Peaks in the power density spectrum that are associated with alpha activity typically observed in the 9–13 Hz range in adults can be seen clearly between 6 and 9 Hz in one-year-old infants (Stroganova et al., 1999) and are lower still in younger infants (Marshall et al., 2002). We also know that infant EEG tends to show greater power at lower (<6 Hz) frequencies and that during development there is an observable increase in power at higher frequencies (Marshall et al., 2002). Whilst these differences have been observed in scalp level EEG data and not at a source level, this evidence highlights differences in the distribution of power at lower frequencies and the overall composition of the 1/f power density curve for infant vs adult EEG.

There is also evidence to suggest that the topographical properties of infant EEG differ from those typical of adult EEG. For example, we know that infant alpha activity projected onto central scalp electrodes is present only in later stages of infant development, presumably accompanying advances in motor skills (Cuevas et al., 2014), although the sources of these scalp activations are yet to be identified. Further, at the source level, infant EEG is often more bilaterally symmetrical than adults (Piazza et al., 2020), although strong topographical asymmetry or localisation to a specific topographical point can be a good indication of artifactual source components (Chaumon et al., 2015). This evidence highlights that infant EEG source components do contain topographically distinct properties to those of typical adult EEG. Overall, the evidence highlights the differences in the spectral and topographical properties between adult and infant EEG both at the scalp and source level. Given how important the spectral and topographical properties are for the classification of ICA components (e.g., Chaumon et al., 2015; see also appendix B, SM Fig. 1) it should be clear from reviewing these studies that attempting to classify infant ICA components using training data from adult EEG would lead to sub-optimal results.

### 1.5 Current study: motivation and goals

In this study, we examine the performance of MARA when applied to samples of 32-channel infant EEG data acquired during naturalistic social interactions. We then adapt the MARA system to better fit the characteristics of infant EEG data. We do this in two ways; (1) by adapting the relevant time-frequency properties derived from the ICA used in classification; (2) by retraining the base classifier using data from infant EEG recordings. From here on we refer to the retrained classifier as iMARA.

To validate the performance of iMARA, we first looked at the inter-rater agreement of ICA components between three expert hand coders. We then compared MARA and iMARA to the validated, manually labelled infant ICA components across two validation studies (classifier validation 1and 2), both using different datasets. Finally (classifier validation 3), we looked at ERP data generated using the different methods to examine in greater detail their ability to remove specific types of artifact.

## 2 Methods

### 2.1 Ethics statement

This study was approved by the Psychology Research Ethics Committee at the University of East London. Participants were given a £ 50 shopping voucher for taking part in the project.

### 2.2 Participants

The same experimental paradigm was used for all validation datasets, but recordings were taken from different sessions (weekly sessions 1 and 8 as part of a broader, 8-week programme of research).

Dataset 1 (Validation 1), 44 healthy (23 F, 21 M) infants participated in the study along with their mothers. Infants were aged 10–12 months (mean 10.72 months, std=1.31). Dataset 1 was taken from the infant’s visit 1 data.

Dataset 2 (Validation 2), 25 healthy (12 F, 13 M) infants contributed data. Infants were aged 10–12 months (mean 12.60 months, std=1.27). Dataset 2 included the same infants with data taken from visit 8.

Dataset 3 (Validation 3), 36 healthy (17 F, 18 M) infants contributed data. Infants were aged 10–12 months (mean 10.70 months, std = 1.08). Dataset 3 is a subset of dataset 1.

### 2.3 Experimental set-up and procedure

Infants were positioned immediately in front of a table in a highchair. Adults were positioned on the opposite side of the 65 cm-wide table, facing the infant. Adults were given toys to play with across a tabletop and asked to “play with their infant as they would normally do at home”. Adults were also asked to lower the volume of their vocalisations to reduce the level of speech-related contamination in the EEG. Dual EEG was continuously acquired from the parents and infants for the approx. 25 min duration of the play session. For this study, we used only the infant’s EEG.

### 2.4 EEG data acquisition

EEG signals were obtained using a dual 32-channel Biosemi system (10–20 standard layout). EEG was recorded at 512 Hz with no online filtering using the Actiview software.

### 2.5 EEG artifact rejection and pre-processing

A fully automatic artifact rejection procedure was adopted, following procedures from commonly used toolboxes for EEG pre-processing in adults (Mullen, 2012, Bigdely-Shamlo et al., 2015) and infants (Gabard-Durnam et al., 2018, Debnath et al., 2020). This was composed of the following steps: first, EEG data were high-pass filtered at 1 Hz (FIR filter with a Hamming window applied: order 3381 and 0.25/ 25% transition slope, passband edge of 1 hz and a cut-off frequency at −6 db of 0.75 hz). Although there is debate over the appropriateness of high pass filters when measuring ERP’s (see Widmann and Schröger, 2012), we aimed to obtain the best possible ICA decomposition. The parameters we used were set up following recent work (e.g., Dimigen, 2020) that examined the removal of eye movement artifacts from EEG data (from a free viewing paradigm) using ICA. Second, line noise was eliminated using the EEGLAB (Delorme and Makeig, 2004) function *clean_line.m* (Mullen, 2012). Third, the data were referenced to a robust average reference (as described in Bigdely-Shamlo et al., 2015). The robust reference was obtained by rejecting channels using the EEGLAB *clean_channels.m* function (using the default settings) and averaging the remaining channels. Fourth, noisy channels were rejected, using the EEGLAB function *clean_channels.m.* The function input parameters ‘correlation threshold’ and ‘noise threshold’ (inputs one and two) were set at 0.7 and 3 respectively, all other input parameters were set at their default values. Fifth, the channels identified in the previous stage were then interpolated back, using the EEGLAB function *eeg_interp.m* (mean 3.3, std, 2.1, min 0, max 9, for dataset 1. Mean 2.2, std, 1.7, min 0, max 6 for dataset 2). In some datasets, channel interpolation reduced the overall rank of the data leading to a fewer number of components than channels as is the norm with ICA. Interpolation is commonly carried out either before or after ICA cleaning, but in general, has been shown to make little difference to the overall decomposition (Delorme and Makeig, 2004). Sixth, the data were low-pass filtered at 20 Hz, again using an FIR filter with a Hamming window applied identically to the high-pass filter. (In the SM we also report a comparative analysis in which data were low pass filtered at 40 Hz instead of 20 Hz (see SM Section 1.5)). Seventh, continuous data were automatically rejected in a sliding 1 s epoch based on the percentage of channels (set here at 70% of channels) that exceed 5 standard deviations of the mean channel EEG power. For example, if more than 70% of channels in each 1-sec epoch exceed 5 times the standard deviation of the mean power for all channels then this epoch is marked for rejection. This step was applied very coarsely to remove only the very worst sections of data (where almost all channels were affected), which can arise during times when infants fuss or pull the caps. This step was applied at this point in the pipeline so that these sections of data were not inputted into the ICA. The average amount of data rejected in this way was 10% (std, 8.7%, min 0%, max 35.6%) for dataset 1% and 6% (std, 5.4%, min 0%, max 20.2%) for dataset 2. Data were then concatenated and ICAs were computed on the continuous data using the EEGLAB function *runica.m.* The mean amount of data entered the ICA was 20.5 min (std 4.7, min 12.9, max 29.7 (mins)) for dataset 1 and 22.3 min (std 4.8, min 13.5, max 32.4 (mins)) for dataset 2. In the raw data condition, we followed the same procedure but without any ICA correction.

### 2.6 Video coding

Video recordings were made using Canon LEGRIA HF R806 camcorders recording at 50fps positioned next to the child and parent respectively. Video recordings of the play sessions were coded offline, frame by frame, at 50 fps. This equates one frame to a maximum temporal accuracy of ~20 ms. Coding of the infant’s gaze was performed by two independent coders. Cohen’s kappa between coders was > 85%, which is high (McHugh, 2012). For our ERP analysis, EEG was time-locked to the onset of gaze/ saccade offline based on the video coding using synchronized LED and TTL pulses.

### 2.7 Hand identification of components for the training set

A full description of how components were identified as containing predominantly neural or artifactual signals by human coders is given in appendix B. Briefly, components were judged first on their topography, second on their power spectrum, and third on their time course, using similar principles to those suggested for adult EEG data (e.g., Chaumon et al., 2015). Components were marked as artifact/ rejected only under the null hypothesis – which in this case is that the component is not considered to contain notable amounts of neural signal. Where a researcher was in doubt over whether a component contained predominantly neural signal we opted to retain that component.

### 2.8 Inter expert reliability

As within any classification system, performance is measured concerning a criterion representing the 'true value' or 'perfect classification'. There exists no gold standard upon which to test any classifier’s performance. As manual classification is the typical approach for ICA data correction (Chaumon et al., 2015) and has been used as a platform to test automatic classification in previous studies (Winkler et al., 2011), we tested the MARA and iMARA systems performance against manual ICA classification. To validate our manual coding, we asked 3 experts to independently rate ICA-components from infant and adult EEG data (SM 1.2, Table S2). We examined whether similar levels of agreement between coders could be achieved for infant ICA components as compared to those in adult data. Results are reported in Section 3.1. Previous research using automated classification methods with adult data from screen-based tasks have reported error rates for inter expert agreement levels of ~10–13% MSE (Winkler et al., 2011).

The measure of performance we use in this study is mean square error (MSE), as has been used in previous automatic classification studies (Halder et al., 2007, Winkler et al., 2011). In its simplest interpretation, MSE is a measure of the error in agreement between systems. For example, an MSE of 0.25 would indicate that the automatic and manual classifiers differed on 25% of the components examined.

### 2.9 Set-up and paradigm for validation dataset 3

In validation 3 we contrasted the different classifiers’ ability to remove stereotypical artifacts from an ERP analysis. This analysis examines event-locked changes relative to infants’ spontaneous gaze shifts during a free-flowing naturalistic interaction. Specifically, we examined moments where infants shifted from looking at a puppet, held at the same height as their mothers face, c 0.10° from the midline (counterbalanced between left and right) to looking at their mothers face, who was always positioned directly in front of the infant. For this analysis, we extracted epochs (mean 39.4, std 12.9) from the continuous data that are time-locked (time 0) to the infants’ fixation onset (saccade onset at −100 ms) (mean 40.8, std 11, min 18, max 64 gaze shifts were included per participant). Evidence from co-registered EEG and eye-tracking studies using free viewing experimental paradigms has shown that when visual responses (e.g., a stimulus appearing on-screen) co-vary with eye movements (e.g., horizontal/ vertical saccades) separation of these signals is possible based on their time and topographical properties (Plöchl et al., 2012). For example, some types of eye movement artifacts e.g., vertical, and horizontal eye movement transients (i.e., only lasting ~200 ms) peak at ~100 ms post saccade onset and have anteriorly dominated topographies, whereas visual processing components tend to peak 100–200 ms after the peak of the artifact and have occipitally dominated topographies (Plöchl et al., 2012). Based on these findings and inspection of our data time-locked to saccade onsets, we set up our comparison in validation 3 between the four cleaning methods described above as follows. For comparison of removal of eye movement artifact time-locked to saccade onset, we compared peak amplitudes of potentials over frontal pole electrodes in the time window −100 (saccade onset) to 100 ms (see also Fig. 3 for visual representation). For comparison of retention of visual response (i.e., the neural signal of interest) we compared peak amplitudes of potentials in the 200–300 ms time window over occipital electrodes. We also compared amplitudes in the 200–300 ms time window over central electrodes to examine how these signals propagated across the scalp. Details of which electrodes were used in each cluster can be found in the supplementary materials section (SM Section 1.3, Table S3).

### 2.10 ERP analysis

Differences in peak amplitude were quantified using the adaptive mean approach. This process involves identifying the peak latency of the ERP potential on a subject-by-subject basis using a broad (100 ms) time window, centered around the time window of interest. For example, in our analysis, we were interested in activity in the −100–100 ms time window. In this case, the adaptive mean approach looks for the latency of the data point with the maximum amplitude + /- 50 ms around the center of the time window (0 ms). Once the peak latency has been identified we took an average of the activity in a 20 ms window around the peak (e.g., as described by Hoormann et al., 1998). This approach is preferred over the more basic comparison of absolute peak amplitudes which would be more susceptible to spurious noise spikes and/or unrepresentative data (Cohen, 2014). All ERP data were baseline corrected using data from the time window −1000 to −700 ms pre gaze onset.

### 2.11 The MARA system for automatic classification of neural/ artifactual components

The MARA classification system identifies artifactual source components from samples of EEG data. For a detailed explanation and the original source code, please refer to (https://irenne.github.io/artifacts/). In brief, Winkler, and colleagues (2011) trained a binary linear classifier to separate neural and artifactual ICA components based on a training dataset of manually labelled ICA components. The comparison between neural and artifactual components was conducted by examining six features derived from the ICA time-frequency properties (see Fig. 1). Here we retrained the MARA system using 617 ICA components from infant EEG data taken from dataset 1 (n = 25 datasets, each contributing on average 25 ICA components). We used a similar feature extraction routine as used by the original classifier, but with a few changes to make it more specific to infant EEG data. For full details see appendix A.

## 3 Results

First (Section 3.1) we validated our manual classification by comparing it with manual classification from two other independent experts. Then, we perform three validation studies to test the performance of iMARA on infant data: first (classifier validation 1, Section 3.2), we tested iMARA and MARA’s agreement with manually classified ICA-components by rater 1. Second (classifier validation 2, Section 3.3), we test iMARA and MARA’s performance on ICA components from an unseen dataset. Third (classifier validation 3, Section 3.4), we examined ERP data generated using the different systems to examine in greater detail their ability to remove specific types of artifact.

### 3.1 Inter-rater validation

To first validate our coding, we asked three experts independently to classify random subsamples of infant (n = 15 datasets, average 25.6 ICs, taken from dataset 1) and adult (n = 15 datasets, average 28.4 ICs, taken from dataset 1) EEG data. Full comparison details are given in SM Section 1.2, Table S2. Between the 3 experts, the average disagreement rate for infant data was 18% (range across three all three experts 14–22%), whereas for adult data it was 15% (range across three experts 12–18%), which is in line with previous reports of human-human error rates for adult EEG data of 10–13% (e.g., Winkler et al., 2011). An independent sample *t*-test revealed no significant differences in the average agreement between adult and infant ICA-components t (14) = 0.98, p = 0.42.

### 3.2 Classifier validation 1

We tested the retrained classifier’s performance against manually classified ICA components from validation dataset 1. This resulted in an averaged MSE between iMARA and the manual classification of 26.59% (sd = 9.93%, range = 54.11%). In comparison, when using the original MARA training data and the original feature extraction routine on dataset 1, the MARA classifier performed with an MSE of 38.35% (sd=15.01%, range = 60.19%). A paired samples *t*-test comparing the percentage of correctly identified components from validation dataset 1 for iMARA vs MARA indicated that MARA had a significantly lower level of agreement with the manual classification than iMARA t (43) = −5.94, p = <0.01. The effect size for this analysis was *d*= 0.92.

### 3.3 Classifier validation 2

**Fig. 2:**
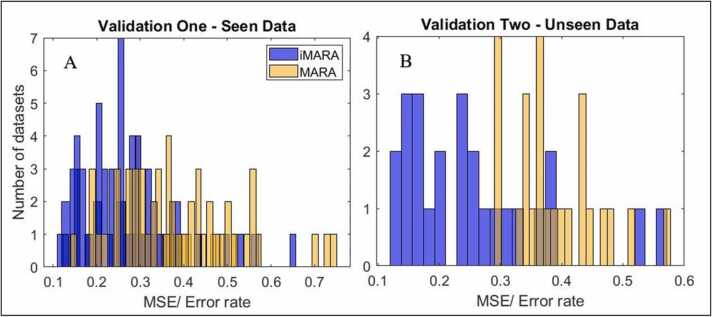
Classification performance for original (MARA) and retrained (iMARA) systems on ‘seen’ and ‘unseen’ data. A) Mean Squared Error (MSE) between original (MARA - yellow) and retrained (iMARA - blue) classifiers and manually classified ICA components for validation one (seen data) for each participant (n = 44) of dataset one. B) MSE between iMARA/MARA and the manual classification for validation two (blind data) for each participant (n = 25) of dataset two. (For interpretation of the references to colour in this figure legend, the reader is referred to the web version of this article.)

We then tested iMARA on an unseen dataset (dataset 2). Classification of the (645) unseen components led to an averaged MSE between iMARA and manual classification of 24.80% (std=8.22%, range=55.43%). In comparison, MARA performed with an MSE of 38.13% (std=8.12%, range=26.63%). A paired samples *t*-test comparing the percentage of correctly identified components from validation dataset 2 for iMARA vs MARA indicated that the original MARA had a significantly lower level of agreement with manually classified ICA components than iMARA t(24) = −4.50, p = <0.01. The effect size for this analysis was *d*= 1.63. (Fig. 2).

### 3.4 Classifier validation 3. Application to ERP study

**Fig. 3:**
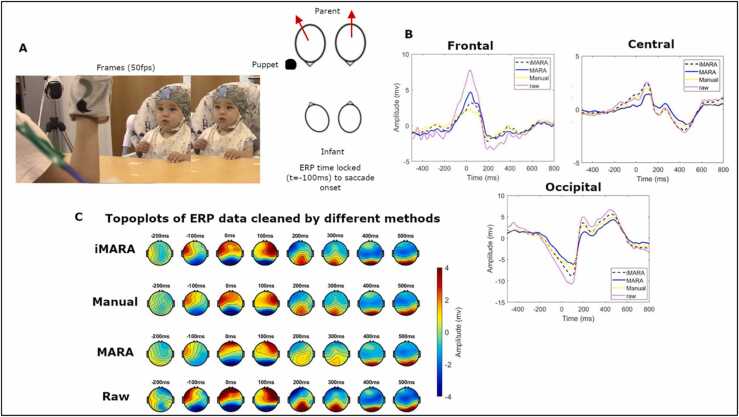
Application of different ICA classification systems to ocular artifact correction in a visual processing ERP study. A) Two-sample frames from which the time-locked gaze shift (−100 ms) were identified, and a schematic showing the experimental set up in which mothers were asked to perform a puppet show with their infants. B) Grand average ERPs over frontal pole, central and occipital scalp regions. Different lines show data cleaned by the different systems, e.g., iMARA- retrained infant classifier, MARA- original classifier, Manual classification, and uncleaned 'raw' data. C) Topoplots of ERP amplitudes, comparing the different cleaning methods to the raw data.

For validation 3 (ERP analysis) we contrasted peak amplitudes (calculated on participant-level data) for each of the four methods of cleaning data (e.g., iMARA, manual cleaning, MARA and ‘raw’) (see Fig. 3). In the SM section 1.7 we present a similar analysis, using time-frequency analyses rather than ERPs. We used the Tukey procedure to correct for multiple comparisons in our ERP analysis. Summary tables for all ANOVAs can be found in SM 1.1, Table S1. Results from the one-way ANOVAs revealed that peak amplitudes for frontal pole ERPs in the −100–100 ms time window were significantly lower for all ICA cleaning methods as compared to the raw un-ICA cleaned data. Peak amplitudes for iMARA were lower than for MARA, indicating that more of the ocular artifact had been removed, but this difference was not significant after correcting for multiple comparisons. For central and occipital ERPs, peak amplitudes for MARA were lower than those observed following manual cleaning and cleaning with iMARA, indicating that MARA had removed more genuine neural data. This effect was significant when examining the relationship between MARA and the raw data, but the difference between MARA and iMARA was not significant after correcting for multiple comparisons (p = 0.10/.11 for central/occipital).

## 4 Discussion

We retrained the popular MARA system for binary (i.e., neural or artifact) classification of ICA-components, to be more sensitive to the types of stereotypical artifacts produced during naturalistic EEG recordings acquired from infants. Our retrained iMARA classifier classified ICA-components from samples of infant EEG with significantly greater levels of agreement with expert manual classification than the original MARA classifier. We examined how well iMARA’s performance generalised to an additional blind dataset as well as its ability to remove ocular-related artifacts in a simple ERP study. Through this, we aimed to provide a tool for developmental EEG researchers wanting to implement automatic ICA cleaning.

### 4.1 Summary of retrained classifier’s performance

In our first validation study, we tested MARA’s and iMARA’s performance against ICA-components manually classified by an expert rater on the full n = 44 dataset. Here iMARA achieved a mean classification error rate of 26% (24% with outliers removed), performing significantly better than MARA (mean error rate for MARA was 38%). In the second validation, we tested iMARA on an unseen dataset, collected using the same experimental setup. In this second validation study, iMARA achieved a mean classification error rate of 25%, again significantly outperforming MARA at 38%. Overall, the differences between iMARA and MARA’s agreement with the manual classification and the inter-rater agreement between humans were marginal (7–8% lower average agreement for automatic classification) relative to the overall error rates of either system (25% MSE for automatic and 18% for manual). This is consistent with the error rates between classifier-human and human-human in previous studies (e.g., 5–6% in Winkler et al., 2011). Our retrained iMARA classifier provides, therefore, a more suitable alternative for classifying paediatric ICA-components than the original MARA system. Additionally, as manual cleaning relies on a large degree of familiarity with ICA and EEG data generally, less experienced researchers using this tool can gain insight into the types of ICA components that are commonly identified as artifacts in paediatric EEG data.

### 4.2 Application of classifiers’ performance in ERP study

We also compared the performance of the iMARA and MARA to manual classification in a simple ERP study. We examined how well each classifier was able to clean the ERP data, focusing on the removal of activity over frontal pole electrodes at the onset of a saccade (gaze shift) and activity over occipital electrodes after a gaze fixation. Our analysis indicated that all methods of ICA cleaning removed statistically similar amounts of frontal pole activity from the raw (un-ICA-cleaned) data, but that neither the data cleaned manually nor iMARA removed all of the frontal pole activity associated with the eye movement artifact. This is consistent with previous research on adults, which found that standard ICA cleaning methods do not entirely remove all frontal EEG activity associated with eye movement artifacts (Plöchl et al., 2012). This is an important point which should be borne in mind in interpreting the results of EEG studies.

Results of validation 3 also show that the post-fixation (gaze onset) visual responses (ERPs) were lower in data cleaned using MARA than for the other types of cleaning, indicating that, while the original MARA classifier did successfully remove comparable amounts of the ocular artifact, it also removed significant amounts of the visually evoked potential (neural signal of interest). This is supported by further analyses (see SM Section 1.4, Table S4) which showed that on average MARA removed 64% of components compared to iMARA which removed 39% suggesting that MARA removed more of the total EEG variance. This effect was observed less strongly in the iMARA group, indicating that iMARA had retained more of the original signal than MARA, but this effect was not significant after correcting for multiple comparisons.

### 4.3 Limitations of the current study

There are two explanations for the higher error rates obtained in this study, compared with the performance of the MARA classifier in the original paper, which was based on adult data (Winkler et al., 2011). First, the classification of ICA components is notably poorer when applied to lower density electrode montages. In a follow-up study, Winkler and colleagues (2014) found using the original MARA classifier that classification error rates increased from 9% to 32% when comparing 104–16 channel electrode setups (although for 32 channel setups it was still comparably lower ~13%) (see also SM section 1.6). This is likely due to the worsening performance of the current density norm feature (a feature estimated from the topographical properties of the data which indicates the source of the activity of the component, see appendix A for further details) with lower density setups as this feature relies on estimations of source activity and use of algorithms that are generally only recommended and applied on higher (>64) density electrode setups.

The second reason for the poorer performance compared to previous applications could be due to the increased ambiguity when classifying ICA-components from infant compared to adult EEG. This may be one of the reasons why ICA is not as widely applied within paediatric EEG research as it is within adult EEG research. In our data, we found that averaged across multiple independent coders, infant source components could only be classified with an inter-coder error rate of 18%, compared with 15% for adult data. Similar rates were also achieved when we asked the same coder (coder 1) to classify the same samples of ICA-components at a later time point. Here the agreement between coder 1 (first and second time rating the same 384 infant ICA components) was 17%. Therefore, we suggest that ICA-components from infant EEG (particularly recorded using naturalistic paradigms) are fundamentally more ambiguous because they are more likely to contain a mixture of neural and artifactual signals, and thus are more difficult to classify binarily.

Components that contain a mix of neural and artifactual signals are also likely one of the main contributing factors for why iMARA ‘mislabelled’ some components. We examined whether there were commonalities in the types of components mislabelled by iMARA (see SM 1.8 and Figs S3 and S4). From visual inspection it didn’t appear that iMARA was systematically mislabelling certain ICA components (i.e., with particular time-frequency and topographical characteristics) as either ‘neural’ or ‘artifact’, compared to the manual labelling. We did observe that as expected the types of components that were commonly being mislabelled (in both directions) tended to contain a mix of neural and artifactual signals and so were ambiguous even the expert human coders.

One limitation of the iMARA system is that the training data used was low pass filtered at 20 Hz and so does not include examples of artfiact contaminated data beyond 20 Hz. As the original MARA system was trained on adult data low pass filtered at 40 Hz we performed additional analysis to examine the performance of MARA on a 40 Hz filter infant EEG dataset (see SM 1.5). For this dataset, we found that both MARA and the human labelling classified over 90% of the components as artifiact, and whilst the agreement between MARA and the human labelling was fairly high (23%) it should be clear that any method that is removing over 90% of the total variance it is also removing large amounts of genuine neural activity. The high rejection rates for manual and automatic classification here are likely due to poor ICA decompositions. This is likely the result of increased muscle artifact contamination, which we know entirely overlaps with the EEG activity in the (~20–300 Hz) spectral range (Muthukumaraswamy, 2013). Here to compensate for this and improve the ICA decomposition we have low filtered the data between 1 and 20 Hz and subsequently limited iMARA’s performance on EEG data beyond this frequency range. Future research may want to add additional components to the existing iMARA training dataset that include examples of components with activity at higher (20 +) frequencies, assuming that these ICA decompositions can be better optimised with infant EEG.

### 4.4 Recommendations for future research

Future research might explore the iMARA’s ability to separate neural and artifactual signals at different frequencies. For example, In SM 1.7 we explore the time-frequency properties of the ERP-responses shown in classifier validation 3. From these plots, it is clear that both classifiers are removing (with varying success) signal that is broadband (i.e., not frequency specific). This may be interesting for future research to explore as eye movements are commonly characterised in time or topographically, but are less often characterised in time-frequency space. Having a clear picture of how ocular artifact in naturalistic data manifests in time-frequency space, as well as, having appropriate tools to identify/ remove it will be of high value to the field going forward. Additionally, it might be useful for future research to integrate iMARA as part of a fully automated EEG pre-processing pipeline either especially for paediatric EEG data or one that is flexible to adult and/or paediatric EEG data.

## 5 Conclusions

This paper presents an automatic ICA classification tool that was specifically tailored to work with infant EEG datasets and EEG data collected during naturalistic parent-infant interactions. We show that the retrained iMARA classifier achieved low classification errors and was better at cleaning stereotypical artifact from a simple visual attention ERP study than the original MARA, adult-trained classifier.

## Declaration of Competing Interest

The authors declare that they have no known competing financialinterestsor personal relationships that could have appeared to influence the work reported in this paper.

## Data Availability

Non-identifiable data (e.g., EEG data) is available from the corresponding author upon request. Identifiable data (e.g., video recordings) may be made available following further permission from the participants.
