# Supplementary material for: Automatic classification of ICA components from infant EEG using MARA
Source: Dev Cogn Neurosci. 2021 Oct 20;52:101024. doi: 10.1016/j.dcn.2021.101024 (PMC8556604; doi:10.1016/j.dcn.2021.101024)
Supplement: Supplementary file 1 — Supplementary material [file mmc1.docx]

**Automatic classification of ICA components from infant EEG using MARA**

**Supporting Materials**

**Appendix A**

*Feature selection used in classification*

In the original paper, the following six features were selected for use in the MARA system. These were originally chosen through an embedded feature selection process (e.g., integrated as part of the learning algorithm) whereby the authors obtained rankings of importance/ effectiveness of 38 different time/frequency/spatial features of the data (for more details see Winkler et al., 2011). This revealed that inclusion of additional features (beyond the six included) did not increase classification performance.

The following two features relate to the component spatial distribution:

Current Density Norm (CDN) - estimation of source position of a component concerning x,y,z spatial coordinates. This process involves dipole fitting the source components (using functionality contained with EEGLAB) and applying an appropriate forward head model (we considered 2142 locations arranged in a 1 cm spaced 3D-grid) and seeking the source distribution with minimal l2-norm (i.e., the ‘simplest’ solution, Winkler et al., 2014).

Components with a high CDN indicate likely artifact. For example, on Figure 1a) component two, three and four all have a relatively high CDN score. These can be compared with components one, two and three of Figure 1b, which all have a relatively low CDN score and were classified as neural. This feature was unchanged from the original study.

Range Within Pattern - the absolute difference between the minimum and maximum of a component’s pattern (spatial distribution) - i.e., how localized the activation is to one position/ electrode. Comparing components two and four in Figure 1a and 1b, we see that artifactual components have a relatively higher range within pattern indicating that these sources are more localized to a singular point, which is taken as an indication of an artifactual component to the classifier. This feature can arise, for example, from poor contact between the surface of the electrode and the scalp. This feature was unchanged from the original study.

The following two features relate to the component-time series:

Mean Local Skewness (MLS) - the mean absolute local skewness of an ICA-component time series, taken in a 1 and 15s (two separate features) sliding time window and then averaged. The idea being that blink components for example would contain epochs with very high amplitude data. This data would be more skewed than a typical alpha generator in which you would expect amplitude to be comparatively unchanged across epochs.

For example, comparing components one, two and three in Figure 1a and 1b, we see that a relatively high MLS indicates artifact, as this component’s time series might contain more high amplitude noise spikes than components with a low MLS. High MLS might arise from faulty electrodes but is also an indication of an ocular motor artifact. For example, in Figure 1a component one, a stereotypical blink component has a relatively high MLS and contains frequent high amplitude spikes in the time series. This feature was unchanged from the original study.

The following two features relate to the component spectral distribution:

Lambda and Fit Error- the deviation of a components power spectrum from a pseudo 1/frequency curve, created by three points of the log spectrum: (1) value at 2 Hz, (2) local minimum in the band 5- 13 Hz, (3) local minimum in the band 33-39 Hz. The spectrum of muscle artifacts, characterized by unusually high values in the 20-50 Hz range, is thus approximated by a comparatively steep curve with high lambda and low fit error. Lambda and fit error are independent features; whereas lambda is a measure of the deviation from the pseudo curve just in the alpha and beta ranges (i.e., steepness of transition between the two), fit error is a measure of the deviation of the components 1/f curve from the entire pseudo 1/f curve between delta to beta.

For example from component two in Figure 1a and 1b, we can see that low lambda (i.e., a less steep curve between alpha and beta) indicates a neural component, whereas high lambda (i.e., a steeper upward curve between alpha and beta) indicates artifact. We can also see that fit error does not always distinguish well between neural and artifactual components in these examples. This is because a neural component with a high alpha peak and an artifact component with a steep upward curve between alpha and beta would both give a high fit error, which can make classification using fit error alone difficult. We adjusted the frequency features to better fit the characteristics of infant EEG data. For fit error instead of taking values at 2hz, 5-13hz and 33-39hz as used in MARA, we take values at 2hz, 5-9hz and 12-19hz. Further for lamda instead of comparing activity in the 8-15hz range to the pseudo 1/f curve as used in MARA, we compared activity in the 6-13Hz to the pseudo 1/f curve.

Alpha Power – The average log band power of the alpha band (8–13 Hz).

From components one and four in Figure 1a and 1b, we can see that high alpha band power indicates a neural component, whereas low alpha power indicates artifact. Instead of taking a value for alpha power in the 8-13hz range as used in MARA, we take a value for alpha power in the 6-9hz range.

**Appendix B**

*ICA rejection criteria*

The criteria used for manual ICA classification for infant EEG data were highly like the principles suggested for adult EEG data (e.g., Chaumon et al., 2015). Components were marked as artifact/ rejected only under the null hypothesis – when the component is not considered to contain notable amounts of the neural signal. Where a researcher was in doubt over whether a component contains real EEG (neural) we opted to retain that component.

All selection of components was performed using the interface provided by EEGLAB’s *pop_selectcomps* function. Often, EEG researchers only reviewed the first ~10 components, as later components account for little overall variance. The machine-based classifier, however, reviews all components on an individual basis, and so for appropriate comparison coders were asked to rate all components on an individual basis.

Components were judged first on their topography, second on their power spectrum, and third on their time course, according to the flow chart below:

*Fig 1. Flow diagram of human ICA classification:*
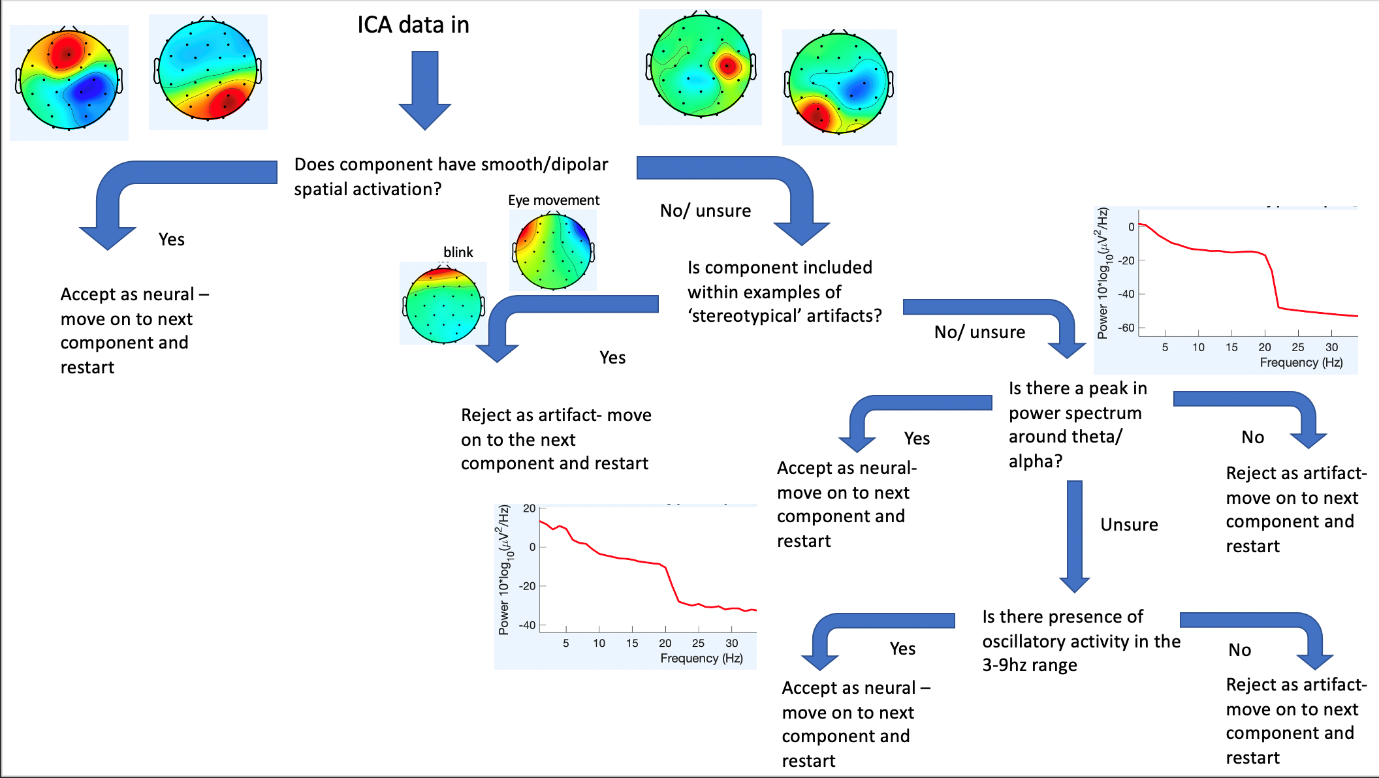


*Stage 1. Evaluation of topographical activation*

Neural components were largely identified by the presence of smooth, dipolar spatial activity. These components were often immediately identifiable in the *pop_selectcomps* component overview and did not need further investigation. A variety of different criteria based on topographical activation were applied:

*Localisation to one electrode.* Components with very localised topographical (i.e., localised to one electrode) activations were always a cause for further investigation (in these cases, coders would move to stage 2). For example:


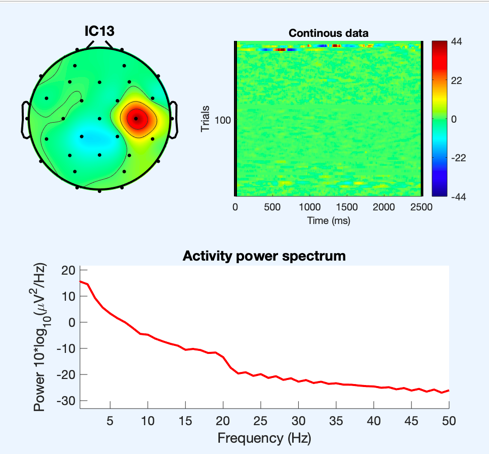


*Frontal peripheral activation.* Certain stereotypical artifactual components were readily identifiable from their topographical maps. For example, components with strong peripheral activations and in particular components with strong/ very localised frontal pole activation (blinks). For example:


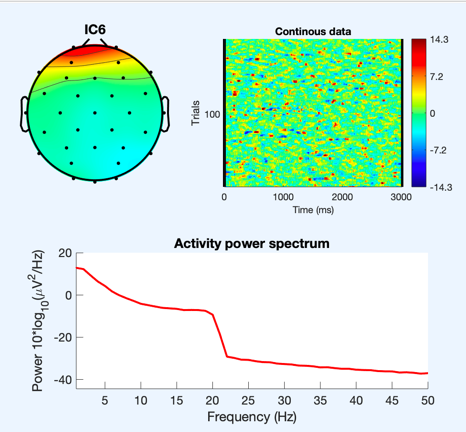


*Bilateral frontal topography.* Components with opposite bilateral frontal topography often indicated horizontal eye movements. For example:


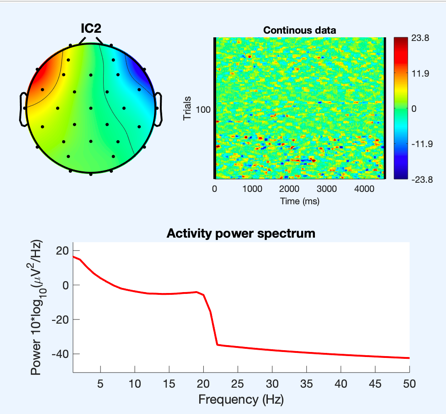

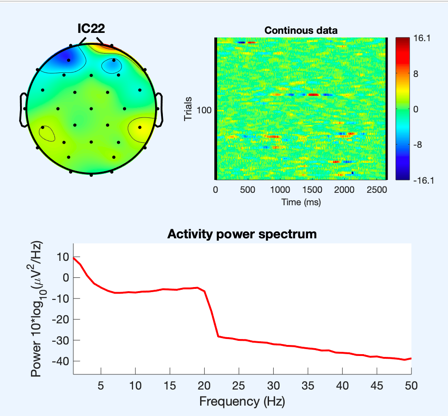


*Temporal peripheral activation*. Components with strong/ very localised temporal activation often indicated jaw/ speech related artefact. For example:


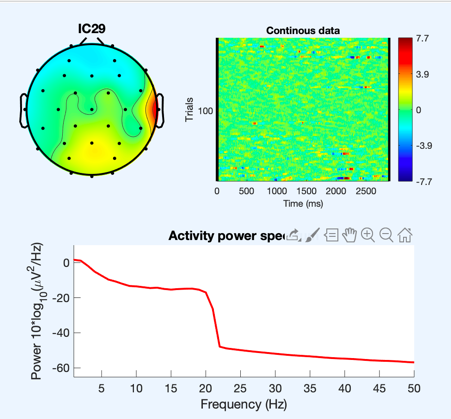

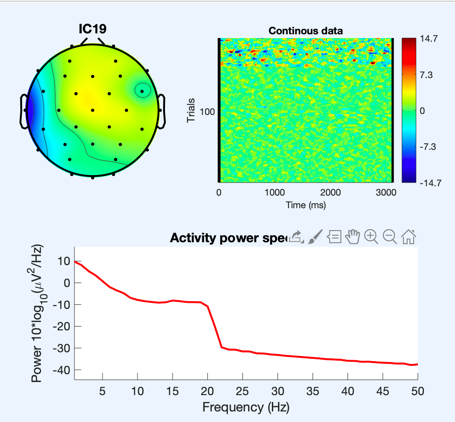


*Activation around P7/P8*. Components with bilateral/strong activation around P7/ P8 (on 10-20 32 channels layout) often indicated neck movement. For example:


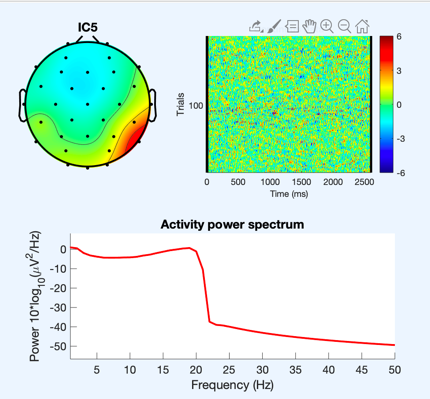

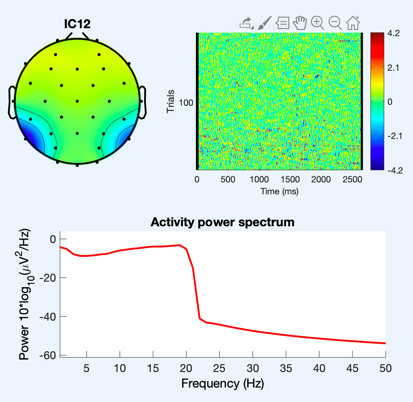


*Stage 2. Evaluation of the power spectrum*

Neural components were identified by 'peaks' in the power spectrum in theta (3-6Hz) and/ or alpha (6-9Hz) range. For example:

 
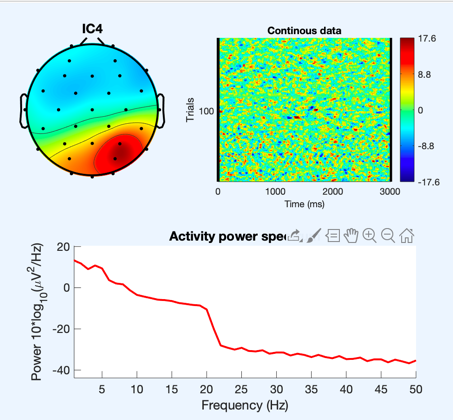

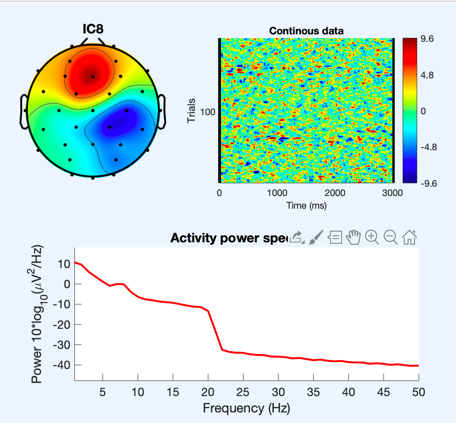


Components with more mixed neural and artefact sources with more subtle peaks in theta/alpha were also identified at this stage as neural. For example:


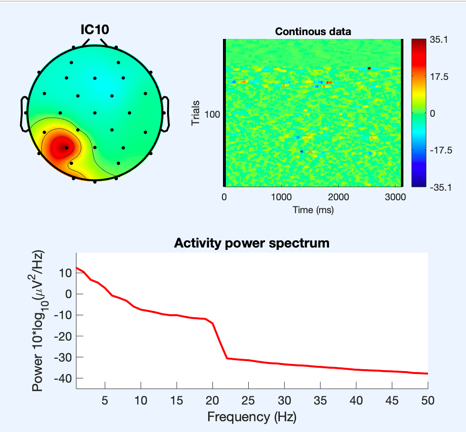

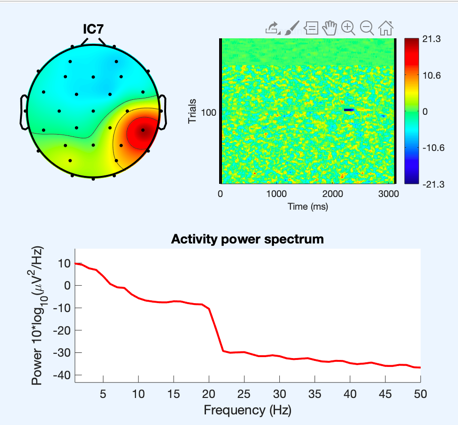


Artefactual components were predominantly identified at this stage through lack of alpha/ theta peak. Further, if a component had no clear 'peak' in alpha but accounted for a lot of total variance coders would move to stage 3 (see below).

Components with power at high frequencies (muscle/ speech artifact) were also marked as artifact at this stage (for examples see the earlier examples for speech and movement-related artifacts).

Components with activity beyond 20Hz were also excluded as the current dataset is low pass filtered at 20Hz – see example below under infrequent high amplitude noise spikes.

*Stage 3. Evaluation of time course*

Artifactual time courses were identified predominantly from their ERP image top right of below figure. If time course activation was mainly driven by infrequent high amplitude spikes (spots of extreme dark colour in ERP image) then it was marked as artefact. For example:


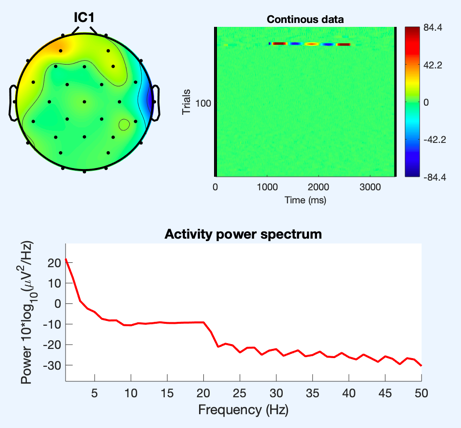


A segment of the component time course:


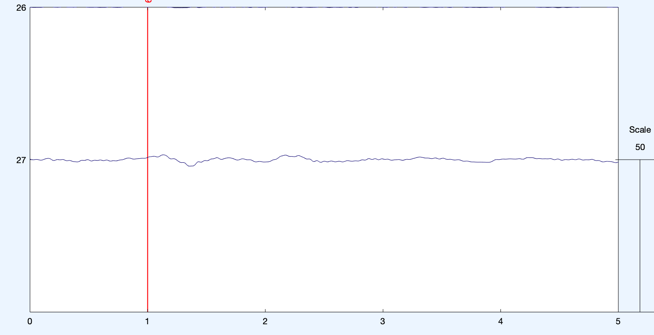

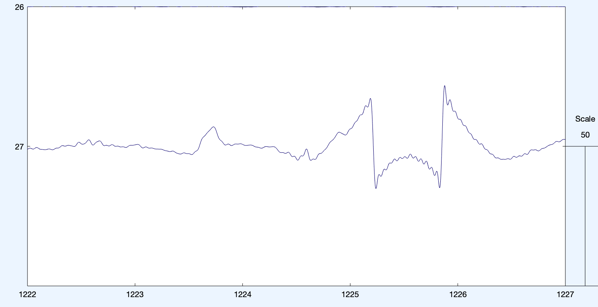


This is different from the example component below as this components time course is predominantly flat throughout.

Other components showed ‘high’ theta/alpha power but no peak. For example:


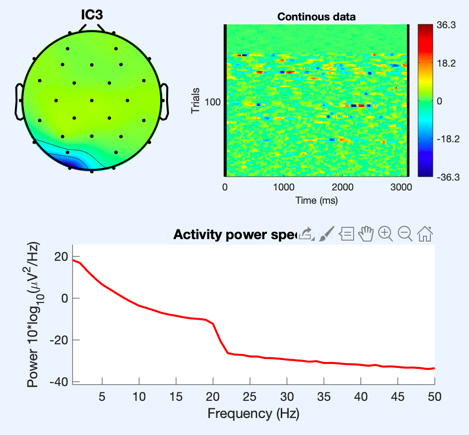


A segment of the component time course:


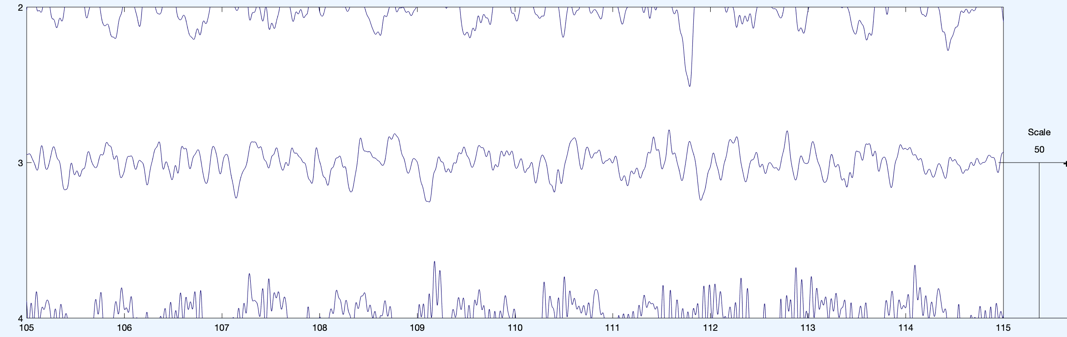


**1. Supplementary Materials**

*1.1 Validation 3: Results of ANOVAs*

*Table S1 summary results of One-Way ANOVA for each scalp region. For frontal pole electrodes, peak amplitudes were compared in the -100 to 100ms time window. For central and occipital components, amplitudes were compared in the 200 to 300ms time window. Electrode groupings used are shown in Table S3.*

| Group ID | | ‘F’ | | | ‘*p’* | | |
| --- | --- | --- | --- | --- | --- | --- | --- |
|  |  | Frontal | Central | Occipital | Frontal | Central | Occipital |
| iMARA | MARA | 2.17 | 2.06 | 6.43**43** | 0.89 | 0.10 | 0.11 |
| iMARA | Manual | 3.92 | 1.54 | 4.39 | 0.85 | 0.70 | 0.88 |
| iMARA | Raw | -2.39 | 0.70 | 1.88 | <0.01 | 0.79 | 0.65 |
| MARA | Manual | 4.74 | 0.57 | 1.39 | 0.42 | 0.60 | 0.41 |
| MARA | Raw | -1.57 | -0.26 | -1.12 | <0.01 | <0.01 | <0.01 |
| Manual | Raw | -3.32 | 0.26 | 0.91 | <0.01 | 0.20 | 0.22 |

*1.2. Inter expert reliability*

*Table S2. Error rates between expert coders on an n=15 subsample of the total data. Each cell shows agreement for infant data (left) and adult data (right)*

|  | Rater 1 | Rater 2 | Rater 3 |
| --- | --- | --- | --- |
| Rater 1 |  | 0.22/0.12 | 0.14/0.15 |
| Rater 2 | 0.22/0.12 |  | 0.19/0.18 |
| Rater 3 | 0.14/0.15 | 0.19/0.18 |  |

*1.3. Electrode positions*

*Table S3. Channel clusters and corresponding 10–20, 32-channel Biosemi positions*

| Clusters | 10-20 Positions | Biosemi 32 channel electrodes |
| --- | --- | --- |
| Frontal pole | Fp1, AF3, AF4, FP2 | 1, 2, 29, 30 |
| Frontal | F7, F3, F4, F8, Fz | 3, 4, 27, 28, 31 |
| Central | C3, CP1, CP5, CP6, CP2, C4, Cz | 5, 6, 8, 9, 10, 21, 22, 23, 25, 26, 32 |
| Temporal | T7, T8 | 7, 24 |
| Parietal | P7, P3, Pz, P4, P8 | 11, 12, 13, 19, 20 |
| Occipital | PO3, O1, Oz, O2, PO4 | 14, 15, 16, 17, 18 |

*1.4. ICA components removed by each method*

*Table S4. Average number and percentage of ICA components removed by each method*

| System | Mean number of components removed (% of total) |
| --- | --- |
| Original MARA | 18 (64%) |
| Retrained MARA | 11 (39%) |
| Hand cleaned | 13 (46%) |

*1.5. 40Hz subsample replication*

All the data used in this study were narrow band filtered between 1 and 20Hz. This was done before ICA correction to improve the signal to noise ratios and the quality of the ICA decomposition. However, in their tutorial ([https://irenne.github.io/artifacts/­](https://irenne.github.io/artifacts/%1f)) Winkler and colleagues (2011) note that application of MARA to narrow band filtered data might result in suboptimal performance, as the spectral features are calculated on the power spectrum between 2 and 39Hz. We, therefore, wanted to test the original classifier’s performance on naturalistic infant data that had not been narrow band filtered. We processed a subsample of 15 datasets in the same way as described in the main text except for the data was low pass filtered at 40Hz this time instead of 20Hz. We then ran ICA, and all source components were again hand labelled by an expert. Results indicated an MSE between the classifier and human labelling of 23% indicating a good degree of similarity. However, the classifier was removing on average 97% of the approx. 32 components, i.e., retaining on average only one source component. The human labelling also removed 90% of the source components, more than double the amount as the data used in the main text. Whilst the MSE is slightly lower than the retrained classifier in the main text, it is clear that this method is far from optimal as clearly if any method is removing over 95% of the total variance it is also removing large amounts of genuine neural activity. The high rejection rates for manual and automatic classification here are likely due to poor ICA decompositions. This is likely the result of increased muscle artifact contamination, which we know entirely overlaps with the EEG activity in the (~20-300 Hz) spectral range (Muthukumaraswamy, 2013). As naturalistic infant EEG data inherently contains more muscle movement (and therefore more artifact contamination) than screen-based paradigms, in the main text we apply a 20 Hz low pass filter to the data to attenuate some of this activity. Comparing the results of the main text with the result obtained when applying the original MARA with data filtered at 40 Hz, it is clear that the 20 Hz low pass filter is greatly improving the ICA decompositions, resulting in a much reduced percentage of components labelled as artifact.

*1.6. MARA adapted strategy*

In their follow up study in which Winkler and colleagues (2014) tested the robustness of their classifier on a variety of novel experimental paradigms and electrode setups, they found that when applied to data with lower density electrode setups e.g. 16 or 32 channels, the classifier’s error rate increased linearly from an MSE between automatic and manual classification from 9 to 32%. This suggests that the classifier performs significantly worse with lower density electrode setups. The reason for this decrease in performance was due to the spatial features performing notably worse (Winkler et al., 2014). The MSE of the CDN feature on 32 and 16 channel setups rose to over 50% compared to 12% with higher density setups. To improve the performance of the original MARA classifier with lower density EEG recordings, the authors proposed an adaptive strategy in which the classifier is subtly adapted to fit the study-specific electrode montage i.e., re-training the classifier on the patterns cut to the specific electrode setup. In their study application of the adaptive strategy with 16 and 32 channel setups lead to an MSE of approx. 16%. We also tested this adaptive strategy on our 32 channel infant EEG data to see whether this led to a similar error rate. We did this by adapting the electrode montage used for spatial feature identification to the Biosemi 32-channel layout but using the same training data as used by the original MARA classifier. This led to an MSE between the adapted strategy classifier and the human labelling of 43%.

Neither the original classifier nor the adapted strategy performed well when applied to our infant EEG data. On one hand, we have a system that achieved decent rates of agreement with hand labelling i.e., MSE of 23% but removes over 95% of the data (e.g., original MARA); on the other hand, the adaptive strategy removes fewer components but has a much higher error rate. Therefore, the final option is to treat the infant data as distinct, retraining the classifier using infant source components and the most salient features for infant EEG.

*1.7. Time-Frequency analysis of ERP data presented in validation study 3*

To further investigate the removal of ocular artifact reported in validation 3. We examined time-frequency responses for the different methods to assess the time-frequency representations of the signal and how this was affected by the ICA cleaning methods. For this analysis single-trial data were first decomposed into their time-frequency representation by multiplying the power spectrum of the EEG (obtained from the fast-Fourier-transform) by the power spectrum of complex Morlet wavelets [*ei*2π*tfe*−*t*2/(2σ2), where *t* is time, *f* is the frequency (which increased from 2 to 16 Hz in 15 linearly spaced steps), and σ defines the width of each frequency band, set according to σ/(2π*f*), σ was set to scale with increases in the centre frequency of the wavelet. We set this parameter to increase logarithmically from 3-10 cycles in 15 increments], and then taking the inverse fast-Fourier-transform. From the resulting complex signal, an estimate of frequency band-specific power at each time point was defined as the squared magnitude of the result of the convolution *Z* (real[*z*(*t*)]2 + imag[*z*(*t*)]2). Power was then decibel normalised to data in the -1000 to -700ms time window.


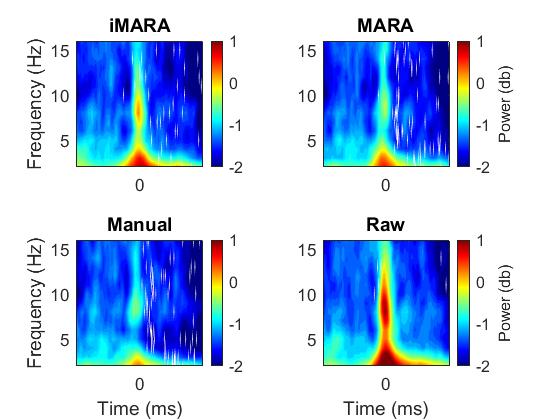

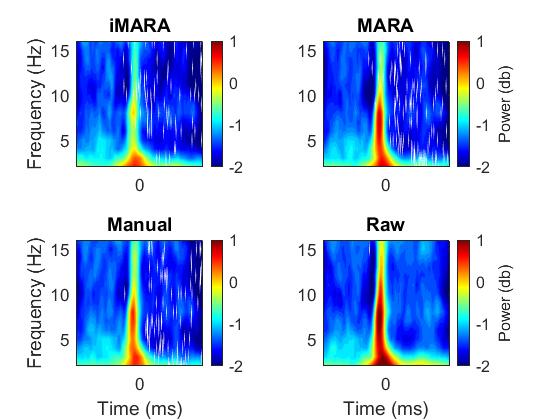


**Frontal Pole TF-Power**

**Occipital TF-Power**

B

A

-2.5 0 2.5

-2.5 0 2.5

-2.5 0 2.5

-2.5 0 2.5

Time (s) (s)

Time (s) (s)

-2.5 0 2.5

Time (s)

-2.5 0 2.5

-2.5 0 2.5

Time (s)

-2.5 0 2.5

Time (s)

Time (s) (s)

Time (s) (s)

*Fig.2 Time-frequency power relative to the onset of infant looks to partner in data cleaned using original 'MARA', retrained 'iMARA’ and manual ICA classification and compared to raw data. A) shows time-frequency plots for data cleaned using 4 methods over frontal pole electrodes. B) shows time-frequency plots for data cleaned using 4 methods over occipital electrodes*

From Fig. 2 we can see that all methods of cleaning resulted in broadband removal of the signal both frontally and occipitally. We can also see that the different methods are to a varying degree removing most but not all the ocular artifact time-locked to the shift in attention. This may be an interesting possibility for future research to explore as commonly eye movements are characterised in time or space but are less often characterised in time-frequency space.

*1.8. Components mislabelled by iMARA*

We performed an additional visual inspection of components that were mislabelled by iMARA to see if they shared any common characteristics. We did this by first looking at components that were labelled as artifact by iMARA, but neural by manual labelling and second looking at components that were labelled as artifact by the manual labelling, but that were classified as neural by iMARA. From visual inspection, we did not observe any strong patterns that distinguished between these two subcategories, i.e., it didn’t appear that iMARA was systematically failing to reject ICA components with certain time-frequency or spatial properties. One thing that we did, however, observe from components mislabelled as artifact by iMARA (as compared to the human labelling) was that they tended to have a ‘high’ range within pattern (RWP). As we have described in appendix A, a high RWP generally indicates artifact to iMARA. From the examples below (Fig. 3) of components mislabelled as artifact by iMARA we can see that all these components have a ‘high’ RWP, but that they also contain some evidence of a real neural signal in the EEG spectra (visual peaks in 1/f curve at theta/ alpha). As the amount of theta/ alpha power is low and the RWP is ‘high’, iMARA classified these components as artifact, but as there is some (albeit subtle) evidence for neural signal the human labelling classified them as neural. We also observed that in some cases in which the component’s time-frequency and spatial properties were completely distorted (see Fig. 4 for examples) likely due to poor connection between the electrode and the scalp, the ‘high’ amount of relative alpha power resulted in iMARA mislabelling these components as neural. This is because ‘high’ alpha power (see appendix A) generally indicated a neural component to iMARA. In future work, we plan to add more components to the training set (currently using just over 600 ICA components) so that the classification system can benefit from a larger and more diverse pool of data.

*Fig. 3. Example*
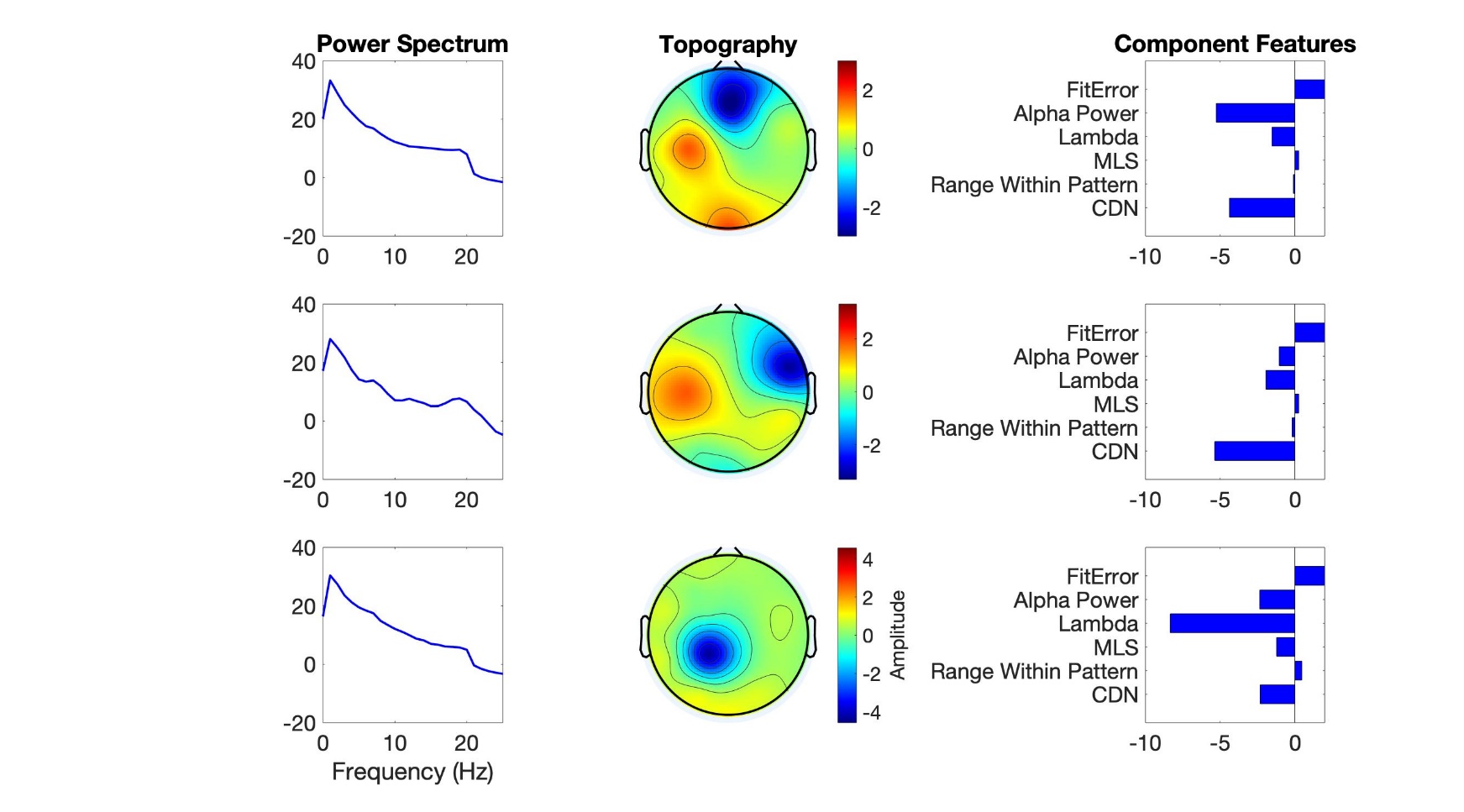
*components ‘mislabelled’ as artifact by iMARA*

*Fig. 3. Example components ‘mislabelled’ as artifact by iMARA. Column one shows the component power spectral density; column two shows the topographical activations; and column three their scores for the six features used in classification. Detailed descriptions of the six features are given in Appendix A.*

*
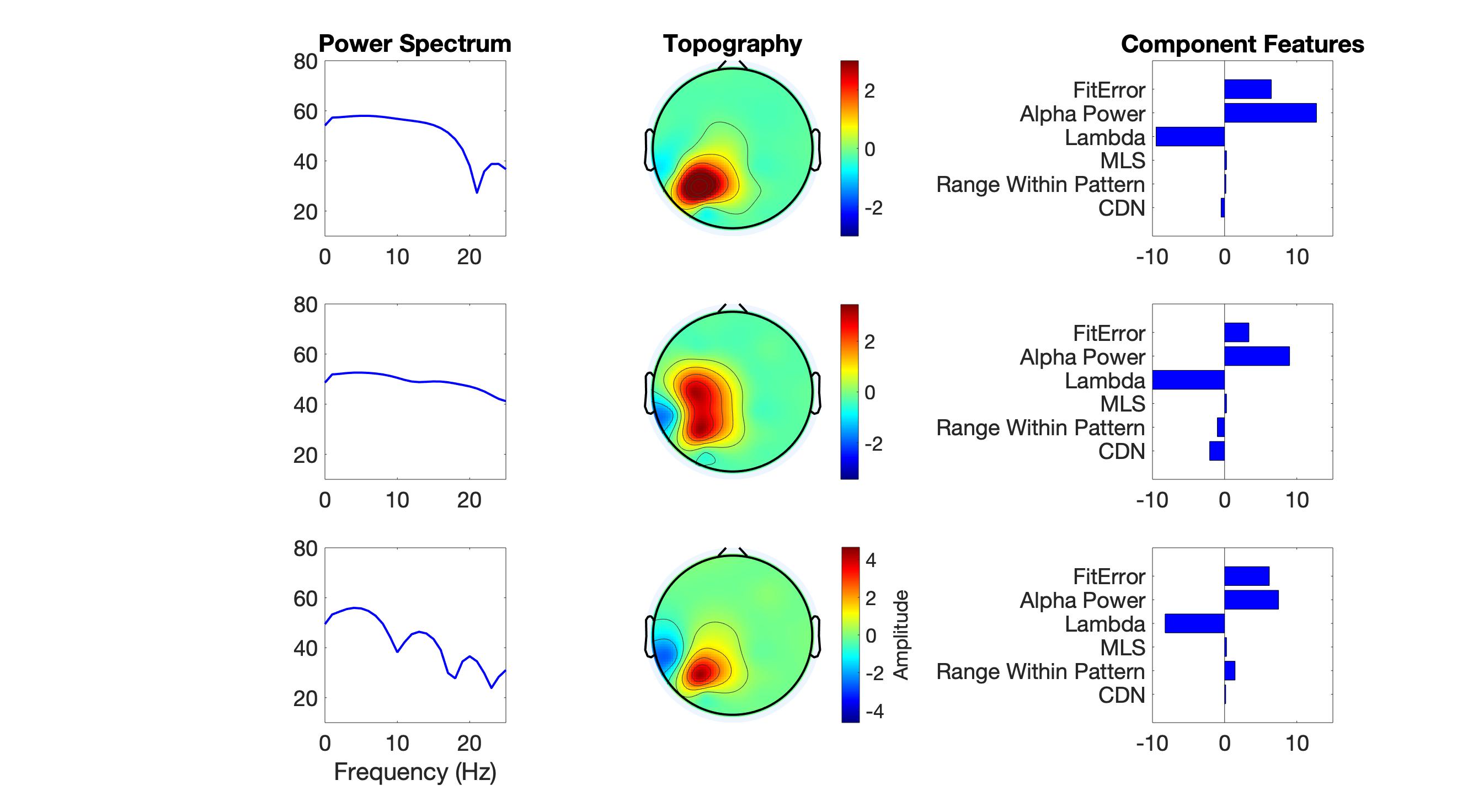
Fig 4. Example components ‘mislabelled’ as neural by iMARA*

*Fig. 4. Example components ‘mislabelled’ as neural by iMARA. Column one shows the component power spectral density; column two shows the topographical activations; and column three their scores for the six features used in classification. Detailed descriptions of the six features are given in Appendix A.*

**References for supplementary materials**

Chaumon, M., Bishop, D. V., & Busch, N. A. (2015). A practical guide to the selection of independent components of the electroencephalogram for artifact correction. *Journal of neuroscience methods*, *250*, 47-63.

Muthukumaraswamy, S. (2013). High-frequency brain activity and muscle artifacts in MEG/EEG: a review and recommendations. *Frontiers in human neuroscience*, *7*, 138.

Winkler, I., Brandl, S., Horn, F., Waldburger, E., Allefeld, C., & Tangermann, M. (2014). Robust artifactual independent component classification for BCI practitioners. *Journal of neural engineering*, *11*(3), 035013.

Winkler, I., Haufe, S., & Tangermann, M. (2011). Automatic classification of artifactual ICA-components for artifact removal in EEG signals. *Behavioural and brain functions*, *7*(1), 30.
